# A specific “at risk” profile related to recent stressful life events in euthymic major depressive disorder

**DOI:** 10.1192/j.eurpsy.2021.313

**Published:** 2021-08-13

**Authors:** G. Serafini, X. Gonda, A. Aguglia, A. Amerio, G. Canepa, P. Geoffroy, M. Pompili, M. Amore

**Affiliations:** 1 Department Of Neuroscience, Rehabilitation, Ophthalmology, Genetics, Maternal And Child Health (dinogmi), University of Genoa, IRCCS Ospedale Policlinico San Martino, Genoa, Italy, Genoa, Italy; 2 Department Of Psychiatry And Psychotherapy, Semmelweis University, Budapest, Hungary; 3 Psychiatry And Addiction Medicine Department, Hôpital Bichat Claude-Bernard, Assistance Publique - Hôpitaux de Paris, Paris, France; 4 Departement Of Neurosciences, Mental Health And Sensory Organs, Sapienza University of Rome, Rome, Italy; 5 Department Of Neuroscience, Rehabilitation, Ophthalmology, Genetics, Maternal And Child Health (dinogmi), Departimento di Neuroscienze, Università di Genova, Genoa, Italy

**Keywords:** Antidepressants, major depressive disorder, family history of suicide, negative distressing/stressful life events

## Abstract

**Introduction:**

Stressful life events (SLE) may influence the illness course and outcome.

**Objectives:**

The present study aimed to characterize socio-demographic and clinical characteristics of euthymic major depressive disorder (MDD) outpatients with SLE relative to those without.

**Methods:**

This sample included 628 (mean age=55.1 ± 16.1) currently euthymic MDD outpatients, among them 250 (39.8%) reported SLE and 378 (60.2%) did not.

**Results:**

After univariate analyses, outpatients with SLE were most frequently widowed and lived predominantly with friends/others. Furthermore, compared to outpatients without SLE, those with SLE were more likely to have a family history of suicidal behavior, manifested melancholic characteristics and higher Coping Orientation to the Problems Experienced (COPE) positive reinterpretation/growth and less likely to manifest a comorbid panic disorder, residual interepisodic symptoms, have used psychiatric medications, and use current antidepressant medications. After regression analyses, having a family history of suicide (OR=9.697; p=≤.05), history of psychotropic medications use (OR=2.888; p=≤.05), and reduced use of antidepressants (OR=.321; p=.001) were significantly associated with SLE. Mediation analyses demonstrated that the association between current use of antidepressants and SLE was mediated by previous psychiatric medications.

**Conclusions:**

Having a family history of suicide, history of psychotropic medications use, and reduced use of antidepressants may confer a specific “at risk” profile related to the enhanced vulnerability to experience SLE.

**Disclosure:**

No significant relationships.

